# The Heterozygote Superiority Hypothesis for Polymorphic Color Vision Is Not Supported by Long-Term Fitness Data from Wild Neotropical Monkeys

**DOI:** 10.1371/journal.pone.0084872

**Published:** 2014-01-03

**Authors:** Linda M. Fedigan, Amanda D. Melin, John F. Addicott, Shoji Kawamura

**Affiliations:** 1 Department of Anthropology, University of Calgary, Alberta, Canada; 2 Department of Anthropology, Washington University, St. Louis Missouri, United States of America; 3 Department of Biological Sciences, University of Calgary, Alberta, Canada; 4 Department of Integrated Biosciences, University of Tokyo, Kashiwa, Chiba, Japan; CNR, Italy

## Abstract

The leading explanatory model for the widespread occurrence of color vision polymorphism in Neotropical primates is the heterozygote superiority hypothesis, which postulates that trichromatic individuals have a fitness advantage over other phenotypes because redgreen chromatic discrimination is useful for foraging, social signaling, or predator detection. Alternative explanatory models predict that dichromatic and trichromatic phenotypes are each suited to distinct tasks. To conclusively evaluate these models, one must determine whether proposed visual advantages translate into differential fitness of trichromatic and dichromatic individuals. We tested whether color vision phenotype is a significant predictor of female fitness in a population of wild capuchins, using longterm 26 years survival and fertility data. We found no advantage to trichromats over dichromats for three fitness measures fertility rates, offspring survival and maternal survival. This finding suggests that a selective mechanism other than heterozygote advantage is operating to maintain the color vision polymorphism. We propose that attention be directed to field testing the alternative mechanisms of balancing selection proposed to explain opsin polymorphism nichedivergence, frequencydependence and mutual benefit of association. This is the first indepth, longterm study examining the effects of color vision variation on survival and reproductive success in a naturallyoccurring population of primates.

## Introduction

The allelic trichromacy of longtomiddle wave sensitive LM opsins in New World monkeys is a textbook example of a balanced polymorphism [1], [2] and one of the few cases where the fitness consequences of variants living in natural populations are amenable to testing. Since its discovery approximately 30 years ago, this color vision polymorphism has intrigued evolutionary biologists and led to extensive debate about its utility in social signaling and finding foods, and about the nature of natural selection behind it e.g., [3]–[5]. Most Neotropical primates possess variable color vision resulting from the polymorphic sexlinked LM opsin gene and a monomorphic autosomal shortwave sensitive S opsin recently reviewed by Jacobs [6]. Females heterozygous for the LM opsin are capable of trichromatic vision, whereas the remaining homozygous females and all males are dichromatic, a condition known in humans as redgreen color blindness [7].

A large and growing body of research has documented 1 that conemediated color vision phenotype can be predicted in a straightforward manner from the opsin genotype, and 2 that there are clear behavioral correlates of variable color vision e.g., [8]–[11]. Yet the evolutionary mechanism maintaining color vision polymorphism remains unknown. In the case of the LM polymorphism, we can rule out drift or random effects with a fair amount of certainty as Hiwatashi etal. [2] documented convincing support for balancing selection on the gene in question. Several hypotheses, drawn from the wider range of evolutionary mechanisms under which genetic polymorphisms are maintained via natural selection [12], have been specifically proposed to explain the maintenance of opsin polymorphisms in primates [3], [4], [13], [14].

**Figure 1:**
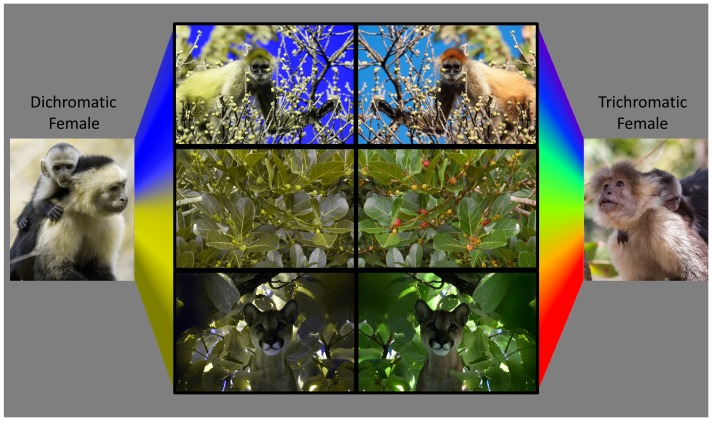
How female capuchins see the world. Color vision phenotype affects perception of relevant objects in the natural environment, including sympatric primates top row, *Ateles geoffroyi*, photo credit F. Campos, ripe dietary fruits middle row, *Ficus ovalis*, photo credit A. Melin and predators bottom row, *Puma concolor*, photo credit N. Parr. Renditions of dichromatic vision left column of images were generated via a computer program customized to simulate primate color vision [55] and were based on the most common dichromatic capuchin phenotype LM allele with peak sensitivity of 561nm.

The most widely accredited explanatory model, the heterozygote superiority overdominance hypothesis, makes the assertion that individuals with trichromatic vision females heterozygous for the LM opsin have a fitness advantage relative to homozygous genotypes, since redgreen chromatic discrimination is useful in foraging for reddish, conspicuous fruits [15]–[18] or young leaves [19] for sociosexual signaling via pelage color [20] or patterns of blood flow [21] or for detecting items of importance in the environment, including sympatric primate species and predators such as tropical felids [22], [23,] Figure 1. Indeed, we have shown that trichromatic females in a population of wild whitefaced capuchins *Cebus capucinus* are more accurate in selecting ripe, reddish fruits than are males or dichromatic females [24]. However, that improved accuracy did not translate to a net increase in feeding rate, perhaps because dichromats used behavioral compensation, had improved color perception in certain light environments or increased their reliance on other sensory modalities, such as olfaction [25]–[27]. Similar results for a sympatric population of polymorphic spider monkeys, documenting no difference in fruit feeding rates between dichromatic and trichromatic monkeys, are reported by Hiramatsu etal. [25], [26], and by Vogel etal. [28] in their study of a neighboring population of whitefaced capuchins.

The other explanatory models niche divergence, negative frequencydependent selection, and mutual benefit of association share overlapping predictions contingent on dichromatic and trichromatic phenotypes each being suited to distinctive tasks [3], [5]. For example, dichromats are reported to excel at breaking camouflage caused by variegated backgrounds [29], [30], which assists them in detecting cryptic predators and prey. Additionally, studies of New World monkeys have shown that dichromatic females and males are more efficient than are trichromats at detecting and capturing camouflaged objects e.g., insects especially under low ambient light conditions [31]–[34]. Divergent abilities due to vision phenotype could therefore allow individuals to specialize on different foods and decrease intragroup feeding competition. Monkeys with rare phenotypes might be especially favored if they experience the least competition for their preferred food type. As social organisms, dichromatic monkeys may also benefit from coresiding with trichromats who lead the group to conspicuously colored fruit trees [18] or who first spot yellowish felids [23]. In turn, trichromats may benefit from capturing camouflaged insects flushed by dichromatic group members [35], or by hearing their alarm calls given to cryptic predatory snakes.

As noted by Cropp etal. [36], it is premature to choose among these adaptationist explanations without studies that examine fitness variability among individuals of different phenotypes. Similarly, Surridge etal. [4] have argued that researchers need to conduct studies to determine whether the proposed visual advantages experienced by trichromats or dichromats actually translate into increased survival and reproductive success. The goal of our paper is to provide such a comparative report on fitness in the trichromatic and dichromatic females of a wild population of Costa Rican whitefaced capuchins. We have previously documented a triallelic LM opsin gene polymorphism in this population, individual members of which exhibit one of three dichromatic phenotypes that are traditionally named after the peak spectral sensitivity of their LM photopigments Red 561nm, Yellow 543nm or Green 532nm, or one of three trichromatic phenotypes GreenRed, GreenYellow, RedYellow [2], [37]. We predict that if the LM polymorphism is maintained via heterozygote superiority, then trichromatic females should have better survival or greater reproductive success than do dichromatic females. Alternatively, if overdominance is not operating and the polymorphism is maintained by an alternate mechanism, then we predict that trichromatic and dichromatic females will have similar overall fitness.

Research on differential reproductive success in wild primates has examined many possible predictors and come to often contradictory results as to the effects of dominance rank, maternal age, infant sex, resource availability and group size on variable fitness of individuals, see review by Pusey [38]. Although age and dominance rank of the mother are often good predictors of reproductive success across many primate species, this is not true in all primate populations and our previous analyses have shown that both maternal age and rank fail to predict female reproductive success in our study animals [39], perhaps because of the confounding effects of frequent aggressive invasions of groups and associated infanticide by immigrating capuchin males [40], [41]. However, the production and survival rates of a female capuchins infants are well predicted by the number of matrilineal kin and by the adult male female ratio in her group higher ratio of resident adult males predicts greater female reproductive success, as well as by the availability of resources and whether or not her previous infant survived past one year of age [42].

In the present study, we build on previous work examining differential reproductive success in primates by testing whether color vision phenotype is a significant predictor of female fitness in wild whitefaced capuchin monkeys. We also build on previous tests of the overdominance hypothesis that found no trichromat advantages in fruit feeding rates [2426], by here providing a conclusive test of whether proposed trichromatic visual advantages translate to enhanced fitness in our study animals.

## Methods

### Ethics Statement

The research reported in this study adhered to protocols approved by the Canada Council for Animal Care through the University of Calgarys Life and Environmental Animal Care Committee LESACC, protocol numbers AC110082, BIO8203, BI 200803, BI 200507, BI 200208, and the University of Albertas Biosciences Animal Care Committee protocol numbers 610151, 151804, 319104. Our research also adhered to the laws of Costa Rica and was conducted with permission from the administrators of the rea de Conservacin Guanacaste and the National Park Service of Costa Rica ACGPI0262013.

We have submitted research protocols on annual applications to the University of Calgarys and the University of Albertas Animal Care Committees and they have approved all the protocols that we used to collect the data analyzed in this study i.e., observational data collection on behavior, births, naturallyoccurring mortality and collection of fecal samples. The analyses in this paper are based on 26 years of life history data births and naturallyoccurring deaths as well as color vision genetics data extracted from fecal samples. Fecal samples are collected noninvasively from the ground below the trees or lowlying vegetation following the defecation of an individual monkey. The monkeys of Santa Rosa are a naturallyoccurring population, freeliving in a national park and no monkeys were handled or interacted with during this study.

### Study Species and Site

Whitefaced capuchins *Cebus capucinus* occur in Central and South America from Honduras at the northern edge of their range down to the northwestern corners of Ecuador and Colombia in the south. They are arboreal omnivores that can survive in a variety of habitats and consume an eclectic diet of fruit, flowers, pith, invertebrates and small vertebrates. They live in multimale, multifemale groups made up of natal females, immigrant males and their offspring. Parkwide censuses of all the capuchin groups in Santa Rosa indicate an average group size of 17 and an overall adult sex ratio approaching 11 [43]. Males disperse from their natal group around the age of 4.5 and continue to move between groups approximately every four years, sometimes alone and sometimes in parallel with their male kin e.g., siblings, cousins [44], [45]. Males sometimes immigrate into groups peacefully when they are not fully grown or when the group has been vacated by the resident males, but most adult and subadult males enter groups by force in coalitions that challenge and drive the prior resident males from the group [41]. During and soon after these aggressive takeovers, many group members are injured and infants often die. Infanticide is the major source of mortality in the first year of life [40].

Females on the other hand, remain in their natal groups, except for a few individuals 12 who occasionally leave with the former resident alpha male when he departs the group, and together they join a neighboring group [46]. Females first give birth at the mean age of 6.5 years and there is little variation around age at first birth 5.9 to 7.3 years 68 of females first give birth in their sixth year of life. Subsequent to first parturition, females typically give birth every two years thereafter unless the first infant dies, in which case the interbirth interval averages 1 year. Female rank does not affect interbirth interval length and dominance rank is not a lifelong characteristic of individual monkeys [39].

The site of our study, Santa Rosa National Park in Costa Rica, was established in 1971 and encompassed 108 km^2^ of tropical dry forest before it was amalgamated in the late 1980s with small neighboring parks and ranchlands into the megapark, rea de Conservacin Guanacaste ACG. The core of ACG remains as Sector Santa Rosa, which is the location of the capuchin groups that we have studied since 1983. Detailed description of this site and species as well as life history and census data on the Santa Rosa capuchins can be found in Fedigan and Jack [43].

### Study Sample and Determination of Color Vision Phenotype

The data analyzed herein come from four closelystudied and contiguous social groups of Santa Rosa capuchins CP, LV, EX and GN on which we have up to 26 years of life history data. These groups have been continuously monitored since their startofstudy dates CP 19832012 LV 19902012 GN EX 20072012 with the exception of seven gaps in CP and LV data collection lasting 26 months each most gaps were two months in length. Birth dates of 15 infants born during these gaps were estimated based on their morphology when we first encountered them and our extensive experience with approximately 140 infants that we have observed closely since the exact day or week of their birth. Death dates of monkeys that disappeared during the gaps were assigned as the midpoint of the period. It is possible that we missed some infants that were born and died during these short periods when groups were not monitored. When new researchers joined our team, they were trained in the field until they could reliably recognize all individuals, ensuring continuity and accuracy of identity and age assignments. Following this method, we have collected data on wellknown monkeys over their complete lifetimes and up to five generations of females have been observed in CP group.

Thus far, we have tracked the survival and reproductive lives of 101 females living in these four adjacent groups. Of these, we determined the color vision phenotype for 49 females 21 dichromats, 28 trichromats. Fortyeight of these 49 colortyped individuals lived beyond two years of age and were included in the longevity postweaning survival analysis. Thirtyseven females produced infants 17 dichromatic and 20 trichromatic mothers and were included in the reproductive analyses Table 1. Among the trichromatic mothers, the GreenRed phenotype was most common and among the dichromatic mothers, the Red phenotype was most common Table 1.

**Table 1:** Female Capuchin Color Vision Phenotypes, Survivorship, and Production of Infants. ML pigment sensitivity describes the peak spectral sensitivity _max_ of the constituent middletolong wavelength sensitive photopigments red561nm, yellow543nm, green532nm.

Animal ID	Color Vision Phenotype	ML Pigment sensitivityies	Age at Departureyears	Depart Type	of Offspring	mean IBI^a^years	mean IBI^b^ years
BAL	Dichromat	Red	9.55	End of Study	1		
CHA	Dichromat	Red	8.05	End of Study	1		
ED	Dichromat	Red	12.52	End of Study	5	2.34	0.93
KIA	Dichromat	Red	10.56	End of Study	2	2.88	
LIM	Dichromat	Red	22.05	Death	8	2.21	0.67
NEM	Dichromat	Red	8.69	End of Study	1		
NYL	Dichromat	Red	11.11	Death	4	1.83	1.16
PIC	Dichromat	Yellow	9.60	End of Study	2	2.06	
PUM	Dichromat	Red	10.59	End of Study	3	1.95	
SAR	Dichromat	Red	11.88	End of Study	3	1.86	
SER	Dichromat	Red	23.44	End of Study	7	2.26	
SHA	Dichromat	Red	9.71	End of Study	2	3.04	
SHE	Dichromat	Red	3.58	Death	0		
SIM	Dichromat	Red	14.29	End of Study	4	2.06	
TIM	Dichromat	Red	16.51	End of Study	4	1.93	
ZAZ	Dichromat	Red	13.80	End of Study	3	2.56	
ROS	Dichromat	Red	19.37	Death	2	1.49	
RIT	Dichromat	Red	12.88	End of Study	3	1.96	
FAW	Dichromat	Yellow	1.21	Death	0		
QUI	Dichromat	Red	5.55	End of Study	0		
GAI	Dichromat	Yellow	4.36	End of Study	0		
ABU	Trichromat	GreenRed	7.57	End of Study	0		
BLA	Trichromat	GreenYellow	26.75	Death	10	2.05	1.02
CHU	Trichromat	YellowRed	13.30	End of Study	5	2.24	0.90
DOS	Trichromat	GreenRed	20.15	Death	8	1.77	1.01
KAT	Trichromat	YellowRed	23.19	Death	11	1.83	0.88
MAY	Trichromat	YellowRed	6.20	Death	1		
ORE	Trichromat	GreenRed	7.61	End of Study	1		
SAL	Trichromat	GreenYellow	16.72	End of Study	6	1.83	
VEL	Trichromat	GreenRed	6.22	Death	0		
BEA	Trichromat	GreenRed	6.69	End of Study	0		
ARI	Trichromat	GreenRed	6.45	End of Study	0		
PAN	Trichromat	GreenRed	2.35	Death	0		
MIN	Trichromat	GreenYellow	22.88^c^	End of Study	3	2.27	1.69
MAX	Trichromat	YellowRed	19.81^c^	Death	3	1.80	
LUN	Trichromat	GreenYellow	22.88^c^	End of Study	3	2.05	1.84
FLE	Trichromat	GreenRed	15.88^c^	End of Study	3	2.08	
LIL	Trichromat	YellowRed	15.88^c^	End of Study	4	2.67	0.99
PET	Trichromat	GreenRed	13.88^c^	End of Study	3	2.61	
MRS	Trichromat	GreenRed	18.88^c^	End of Study	6	2.21	0.98
PAD	Trichromat	GreenYellow	9.88	End of Study	1		
CHO	Trichromat	GreenRed	9.35	Death	2		0.74
ATH	Trichromat	YellowRed	12.14^c^	Death	3	3.16	
ELE	Trichromat	GreenYellow	13.88^c^	End of Study	3	1.95	
CAL	Trichromat	GreenRed	11.88^c^	End of Study	5	1.65	0.78
HEL	Trichromat	YellowRed	9.88	End of Study	3	2.05	1.00
CRE	Trichromat	GreenRed	2.77	Death	0		
THY	Trichromat	GreenRed	4.47	End of Study	0		
CAS	Trichromat	GreenRed	2.97	Death	0		

aMean of uncensored complete IBIs when the first infant in the interval lived one year of age.

bMean of uncensored complete IBIs when the first infant in the interval diedone year of age.

centered study as an adult or subadult, age estimate based on morphological features at first sighting in 2007.

doi10.1371/journal.pone.0084872.t001

We performed color vision genotyping from fecal DNA collected noninvasively. Multiple 25 fecal samples were collected from all individuals in each social group. Approximately 1gm of feces was stored at ambient temperature in 5ml of ASL buffer QIAamp DNA Stool Mini Kit Qiagen, predispensed into sterile 15ml plastic vials. Fecal DNA was isolated using the QIAamp DNA stool mini kit Qiagen Inc. in a biological safety cabinet. We sequenced the LM opsin genes of each individual from a minimum of two different fecal samples, requiring two identical results to assign a color vision genotype. Entire gene sequences of the three alleles of *Cebus capucinus* were registered in GenBank in 2005 under accession numbers AB193773 P561 allele, AB193778 P543 allele and AB193784 P532 allele. Amino acid residues at the three critical tuning sites exon 3, site 180 exon 5 sites 277 and 285 were determined to assign the color vision genotype [26]. We minimized the chances of allelic dropout by requiring that at least one fecal sample from each female contained no less than 200pg of genomic DNA. Further details, including our PCR and sequencing protocols, are described in previous publications [24], [26].

### Measures of Fitness and Reproductive Success

The rate of infant production fertility and survival of those infants, and longevity of the mother herself, are three important components of female fitness [38], [47], [48]. We address these three variables in turn.

In iteroparous organisms that habitually give birth to one infant at a time most primates, fertility rates depend on the length of time between parturition events, i.e., the interbirth interval, or IBI [49]. We calculated the intervals between live births as our measure of fertility rate. IBIs are commonly used in primatology as a proxy for the number of infants born per female in a given time interval [38], [50], and since our data on births were unavoidably constrained by the arbitrary start and stop dates of our study, it was more appropriate to use IBIs rather than number of infants born as our measure of female fertility. We included in our analyses intervals that were right censored by the stop date of our study or deathdeparture of the mother, since their exclusion may result in systematic bias toward shorter intervals [51]. Furthermore, our previous finding that death of an infant prior to 1year of age shortens the length of the interbirth interval [39] led us to distinguish between intervals in which the first infant died prior to the age of 1year and those in which the first infant survived. By separating out IBIs in which the first infant died prematurely, we were also able to remove any effects of early infant deathinfanticide on IBI length.

To be reproductively successful, a female primate needs not only to produce infants but also to experience high rates of survival in her offspring and to live a comparatively long life herself. We examined the survival of a females infants from their births to two years of age. We used age two as the cutoff point for calculating offspring survival on the assumption that prior to this age, an immature monkeys survival would be primarily a function of their mothers rather than their own color vision phenotype. This is because in the first year or two of life, prior to weaning, a young capuchin depends on its mother for milk and because the mothers ecological fitness and health determine the availability of her milk and the amount of care e.g., transportation and protection she can provide to the infant.

Our second measure of survival was that of the mother herself longevity which we calculated from two years of age i.e. postweaning until her death or the end of our study. Sometimes we find the cadaver of a deceased study animal on the forest floor, or we observe them to be wounded or ill before they disappear, in which case we record them as dead. Females seldom disperse 12 of females have emigrated out of, or immigrated into our study groups and in the few cases where females have dispersed from our study groups, we have tracked them to a neighboring group. Therefore, we assume that any cases of postweaning female disappearances are deaths.

For both IBIs and the survival of infants, we accounted for potential autocorrelations in the fitness events experienced by each particular mother by introducing a random effect of the identity of the mother in the analyses. As noted by Jones etal. [49], introducing the random effect of the mothers identity also provides an indirect measure of phenotypic quality or frailty.

### Statistical Analyses

To assess the pace of infant production, we used mixed effects Cox regressions coxme and coxph procedures [52] in R [53] and analyzed the length of 139 interbirth intervals IBIs, Table 1. There were a total of 101 completeuncensored and 38 censored intervals for 37 mothers of known visual phenotypes. The model included IBI as the dependent variable, a fixed effect of the mothers color vision phenotype dichromatic or trichromatic, a fixed effect of whether the first infant in the interval died prior to age 1, and a random effect of the identity of the adult female. Censored values included cases where the female was still alive at the end of the study but had not yet given birth to another infant and cases where a female had died. Thus, if a female had N births, there were N values for IBI, the first being the interval between births 1 and 2, and the last being the censored interval between last birth and deathend of study.

To determine the effect of the mothers color vision type and of the mothers identity on the survival of her infants, we used mixed effects Cox regressions combining coxme and coxph procedures in R to analyze the survival of infants from birth to age 2. We included all 139 infants born to 37 females with known color vision phenotypes Table 1. The model included age of the infant at death or at the end of the study if the infant was still alive but less than 2 years of age, as well as a fixed effect of the mothers color vision type and a random effect for the identity of the adult female.

To examine the longevity of trichromatic versus dichromatic females, we used a Cox proportional hazard regression the coxph procedure in R to analyze the survival of individual females from the age of two years on. There were a total of 48 females included in this analysis for which we know their color vision phenotype and that they survived beyond two years of age Table 1. The model included a survival function for the females as a function of being trichromatic or dichromatic. The age at death was rightcensored for those individuals still alive at the end of the study. The age of entry into the study was left censored if the individual did not enter the study at birth in which case we estimated her age, based on agerelated morphological features such as brow and nipple length, and based on 26 years of observing the aging process in females of known age.

Because the advantage proposed to exist for trichromats is hypothesized to be particularly strong for those that have maximum sensitivity near the green or red end of the spectrum, we repeated all three analyses IBI, offspring survival, maternal survival, using only reproductive data from GreenRed trichromats and comparing them to dichromats.

## Results

### Interbirth Interval Duration in Trichromatic versus Dichromatic Females

**Figure 2:**
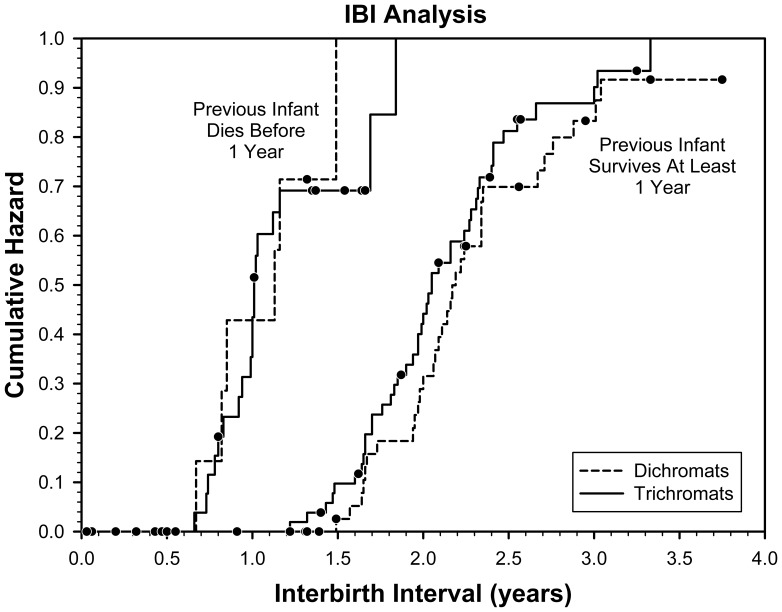
Cumulative Hazard functions for interbirth intervals IBI as a function of time in years for females with dichromat color vision dashed lines and trichromat color vision solid lines and for IBIs in which the first infant in the interval did left lines or did not right lines die at 1year. Cumulative hazard represents the probability that an interbirth interval ends on or before a particular age. Dots represent censored IBIs i.e., those IBIs where the female died prior to the next birth or where at the end of the study period the female had not yet given birth again.

We examined the length of interbirth intervals IBIs N139 as a function of the effect of 1 the mothers color vision phenotype 2 the death of the first infant in the interval at 1year of age and 3 the random effect of the mothers identity. The interbirth intervals of trichromatic versus dichromatic females did not differ significantly ^2^0.445, df1, p0.504, Figure 2. For IBIs where the first infant in the interval lived at least one year, the fitted median IBI was 2.05 years N56 for trichromatic females and 2.19 years N45 for dichromatic females.

In accordance with a previous study [39], we found that interbirth intervals in which the first infant died before age 1 were significantly shorter than intervals in which the first infant survived ^2^59.5, df1, P0.001 and this was true for both trichromats and dichromats Figure 2. For IBIs where the first infant in the interval died before age 1, the fitted median IBI was 1.01 years N28 for trichromatic females and 1.13 years N10 for dichromatic females.

Finally, we found that the individual identity of the mother i.e., the random effect of the mother independent of her vision phenotype contributed significantly to the explanatory power of the model ^2^4.460, df1, p0.035.

### Survival of Infants Born to Trichromatic versus Dichromatic Mothers

**Figure 3:**
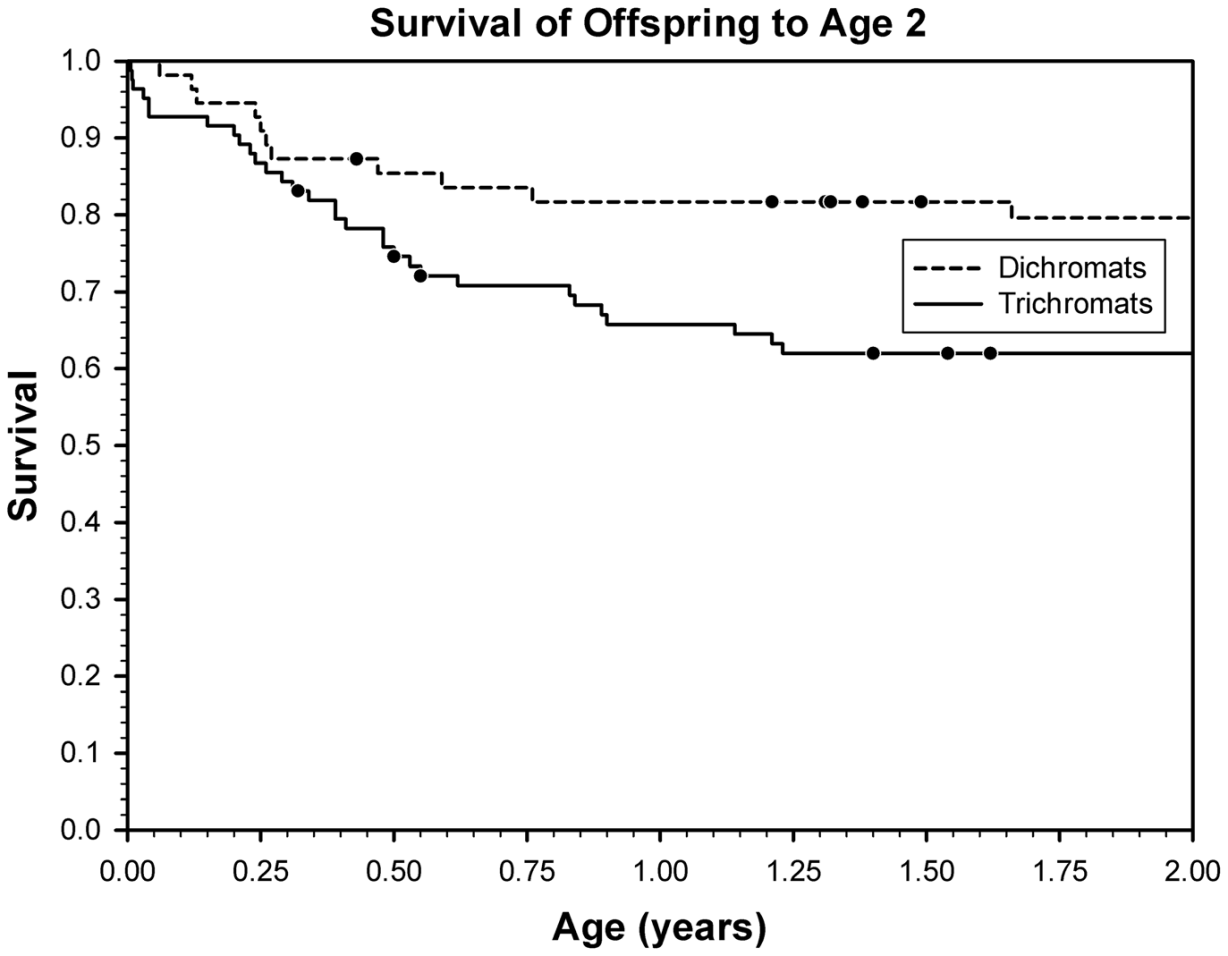
Survival of dependent offspring between birth and age two in years as a function of mothers color vision type dichromat dashed line or trichromat solid line. Dots represent censored observations i.e., those individuals that were still alive and less than 2 years of age at the end of the study.

We assessed the survival of infants N139 from birth to the age of two years, the typical timing of weaning, as a fixed effect of the mothers color vision phenotype and the random effect of the mothers identity. Infants of trichromatic mothers did not live longer up to two years than those born to dichromatic mothers ^2^1.49, df1, p0.221, nor did the identity of the mother contribute to explaining variation in the probability of the offspring surviving to the age of two ^2^0.005, df1, p0.940, Figure 3. For trichromatic females, 61.9 of their offspring survived to age 2, whereas for dichromatic females, 79.6 of their offspring survived to age 2.

### Survival of Trichromatic versus Dichromatic Females Post Weaning

**Figure 4:**
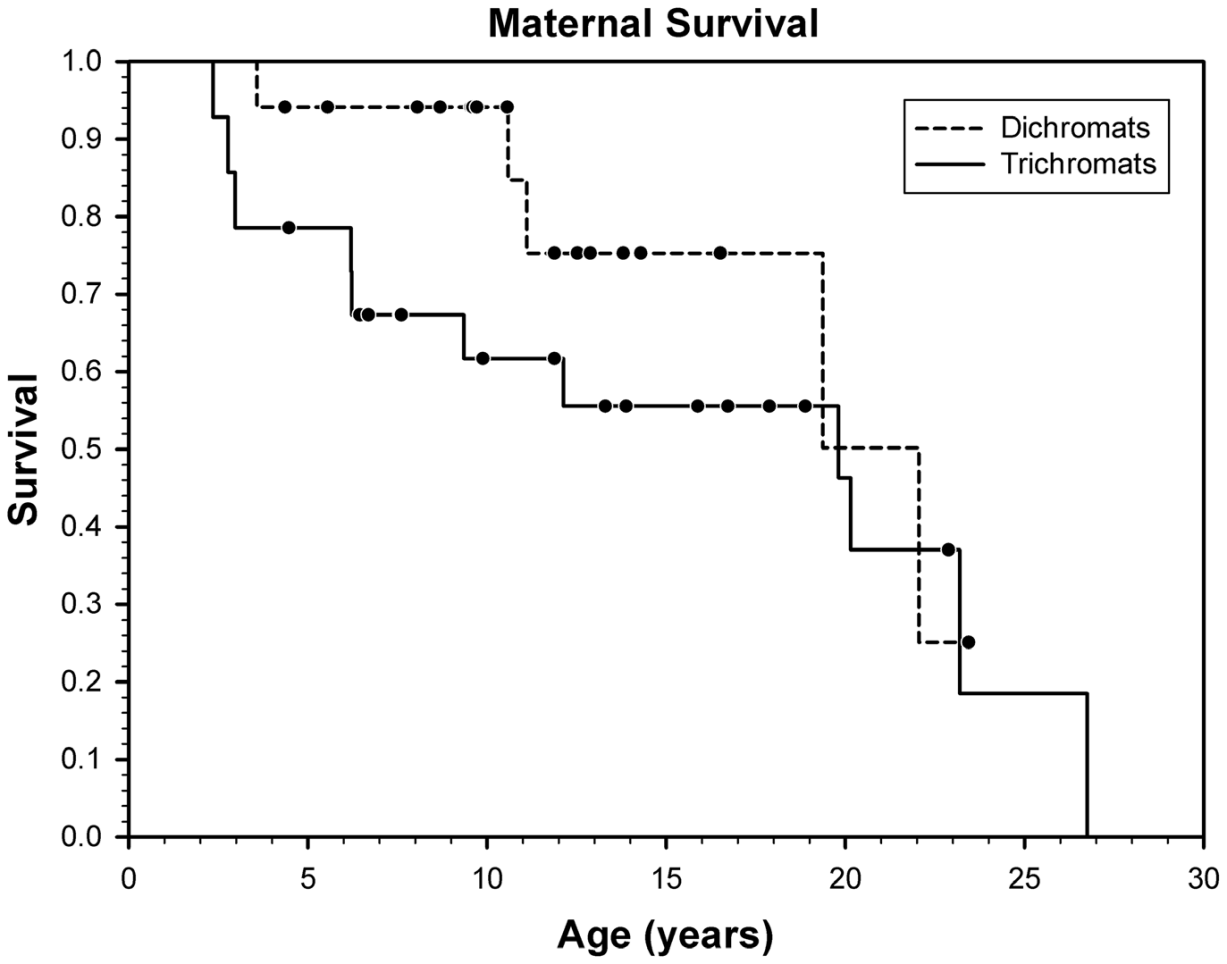
Survival of females from 2 years of age as a function of their color vision type dichromats dashed line trichromats solid line. Dots represent censored observations i.e., those individuals that were alive and older than 2 years of age at the end of the study.

When we examined the survival of females past the age of 2 years as a function of their color vision phenotype we found that survival did not differ between trichromats and dichromats ^2^0.91, df1, p0.339, Figure 4. The median predicted survival time for dichromatic females was 22.1 years N20 and 19.8 years for trichromats N28. Additionally, if we consider only those females who actually died before the end of our study, we can see from Table 1 that they lived between 1.21 and 26.75 years. The mean age of survival for the trichromatic females who died before the end of the study n11 was 11.99 years and the mean age of survival for dichromatic females who died N5 was 11.46 years.

### Comparison of GreenRed Trichromats to Dichromats for Interbirth Intervals, Offspring Survival and Maternal Survival

Because the LM photopigments of GreenYellow and RedYellow trichromats are less spectrallyseparated equivalent to anomalous trichromacies in humans than the photopigments of GreenRed trichromats, it is possible that the former phenotypes might experience lower fitness and bring down the trichromatic group mean. To test for this, we performed the same analyses as those presented above, but this time we limited the sample of trichromats to the GreenRed phenotype.

We found that the results for all of these analyses were qualitatively identical to those presented above for the trichromatdichromat comparison. There were no significant differences between GreenRed trichromats and dichromats on any of our three measures of female fitness. Specifically, the phenotype of the mother GreenRed trichromat versus dichromat has no discernible effect on the length of her IBI ^2^1.41, df1, p0.234, and the early death of the first offspring in an IBI leads to a shorter IBI for both GreenRed trichromats and dichromatic mothers ^2^34.0, df1, p0.001. Second, the mothers vision phenotype does not affect the offsprings survival up to 2 years ^2^2.54, df1, p0.109. Third, the survival of females after the age of 2 is not significantly affected by their vision phenotype ^2^3.66, df1, p0.055 and the trend is in the direction of dichromats surviving better than the GreenRed trichromats.

## Discussion

Although heterozygote superiority appears from the literature to be a widely accepted mechanism explaining primate color vision polymorphism, we found no significant advantage to trichromats over dichromats for the three measures of fitness we examined in female monkeys. Not only did the differences fail to reach significance, the trends for infant and maternal survival were in the opposite direction than predicted by the heterozygote superiority hypothesis Figures 3 4. The one clear and consistent prediction from the literature is that GreenRed trichromats should experience visual advantages and therefore fitness advantages in comparison to the five other phenotypes found in capuchins, and in particular GreenReds should do better than the dichromats [54]–[60]. However, even when we limited our analyses to the GreenRed trichromat females, there was no indication whatsoever that greenred trichromatic females do better than dichromats on any measure of fitness fertility rates, offspring survival, maternal survival. We suggest therefore that an alternate selective mechanism is operating to maintain color vision polymorphism in our study animals.

Despite the lack of fitness differences due to color vision phenotype, we did find significant variation in IBIs attributable to the mothers identity, indicating that some aspect of phenotypic quality, other than color vision, is influential in the pace of infant production in our capuchins. These results are in accordance with other recent studies on female primates e.g., 49 in which the effect of the mothers identity was investigated. Dominance rank and age are unlikely to explain these characteristic IBI lengths that are consistent over a females lifetime, in particular because rank and age change over the course of a female capuchins life and because our previous studies of the effects of dominance and age failed to demonstrate that these variables are significant predictors of IBI length in our study animals [39]. However, other aspects of a females behavior may affect her lifelong fertility pattern, in that some females may consistently behave in ways to enhance their probability and frequency of producing offspring. For example, they may act strategically to achieve more conceptive matings or to acquire higherquality resources that enhance fertility.

Thus, this study has demonstrated that there is individual heterogeneity in at least one component of fitness in female capuchins length of the interbirth interval but that variability appears not to be driven by a superiority of the trichromatic phenotype, even when we factor out the effects of early infant deaths. Hiwatashi etal. [2] report a clear indication that balancing selection is maintaining color vision polymorphism in these same study animals yet our analyses show that this polymorphism does not result in differential fitness of trichromats over dichromats, which suggests that evolutionary ecologists should turn their attention to deriving testable predictions from the alternate hypotheses for mechanisms of balancing selection, all of which postulate that multiple color vision phenotypes each have their own advantages under distinct ecological or social conditions.

A surprising dearth of attention has been directed towards evaluating the three alternate mechanisms that have been proposed to explain intraspecific opsin polymorphism nichedivergence, frequencydependence, and mutual benefit of association. In part, this may be due to the difficulty of teasing apart these hypotheses due to their largely overlapping predictions. To our knowledge, only one field study has directly addressed an alternate hypothesis. Melin etal. [13] concluded that nichedivergence in diet is unlikely to be operating on polymorphic color vision in Santa Rosa capuchins due to the similarity of diet composition across group members that is driven by cohesive group behaviors. However, the scope of this study was restricted to broad categorizations of food types. A more detailed and nuanced examination of niche differentiation by dichromats and trichromats, including canopy use e.g., [17], [61] and finer dietary categorization, may reveal important differences. Furthermore, future work should investigate the extent of communal resource discovery and use, versus individual detection and monopolization of foods. Finally, it will be prudent moving forward to assess the importance of visual tasks unrelated to foraging, such as predator detection, which may be an important factor under a mutualbenefit of association scenario.

In their review article on linking genotypes, phenotypes and fitness, Bradley and Lawler [11] note that it is an open question as to how shortterm differences in foraging skills of dichromatic versus trichromatic primates translate to differences in ultimate fitness. Our study suggests that the distinctive foraging skills of dichromatic and trichromatic whitefaced capuchins each confer their own advantages such that neither phenotype has an overarching fitness benefit.
